# 

**Published:** 1852-11

**Authors:** 


### CONTENTS

### OF NO. V.

---

### ORIGINAL COMMUNICATIONS.

### ESSAYS AND CASES.

Art. I.—Report on Asiatic Cholera, as it appeared in New Albany, Indiana, in the year 1852. By Albert de Leszczynski, M. D., of New Albany. - - 360
Art. II.—A remarkable case of Reflex Nervous Action. By J. W. Bright, M. D., Louisville, Ky. - - 303
Art. III.—A case of Encysted Tumor Extirpated. By M. J. Bray, of Evansville, Indiana. - - ‘ - 396

### REVIEWS.

Art. IV.—Clinical Reports on Continued Eever. By Austin Flint, M. D., Professor of the Principles and Practice of Medicine, and of Clinical Medicine, in the University of Buffalo, and Editor of the Buffalo Medical Journal 398
Art. V.—Report of the Medical Department of the University of Pennsylvania, for the Session of 1851—to the Alumni of the School. By the Medical Faculty - 417

### SELECTIONS FROM FOREIGN AND HOME JOURNALS.

Fellis Bovini as a Medicine - - - - - - - - 429
Radical Cure of Reducible Hernia, by Injection, . - - 433
Paste made from Pumpkin Seeds in the Treatment of Tænia, 435
Treatment of Hooping Cough, - - - - - - - - 437

Strangulated Hernia in a child five months old; Operation; Recovery, - - - - - - - - - - - 438
Prevention of Salivation, - - - - - - - - - 439
A case of Poisoning with oil of Tansy, - - - - - 440
On the influence of Poisons upon Animal Heat as the cause of death, - - - - - - - - - - - 443
On the normal degree of the Temperature of Man, - - 446
On the Influence exerted upon the General Temperature of the Body by a change in the Temperature of the Extremities 448
Case of Conception before the appearance of the Menses, - 451
Extraordinary Case of Hydrocephalus, - - - - - - 452

ORIGINAL INTELLIGENCE.

Proceedings of the Kentucky State Medical Society, - - 457

